# Single-Photon Emission Computed Tomography in Unilateral Asterixis

**DOI:** 10.31662/jmaj.2025-0350

**Published:** 2026-05-22

**Authors:** Hideyuki Matsumoto, Toshiyuki Kakumoto, Naohiro Uchio, Yasuhisa Sakurai

**Affiliations:** 1 Department of Neurology, Mitsui Memorial Hospital, Tokyo, Japan

**Keywords:** asterixis, negative myoclonus, ischemic stroke, cerebral infarction, single-photon emission computed tomography (SPECT)

An 80-year-old female patient presented with sudden onset of right hemiparesis and right-sided unilateral asterixis. Diffusion-weighted brain magnetic resonance imaging revealed high-signal-intensity lesions located between the posterior limb of the left internal capsule and the putamen (Figure 1). Despite this focal ischemic stroke, 99mTc-ethyl cysteinate dimer (ECD) single-photon emission computed tomography (SPECT) demonstrated extensive hypoperfusion in the left lentiform nucleus, thalamus, and cerebral cortex (Figure 2).

**Figure 1.**
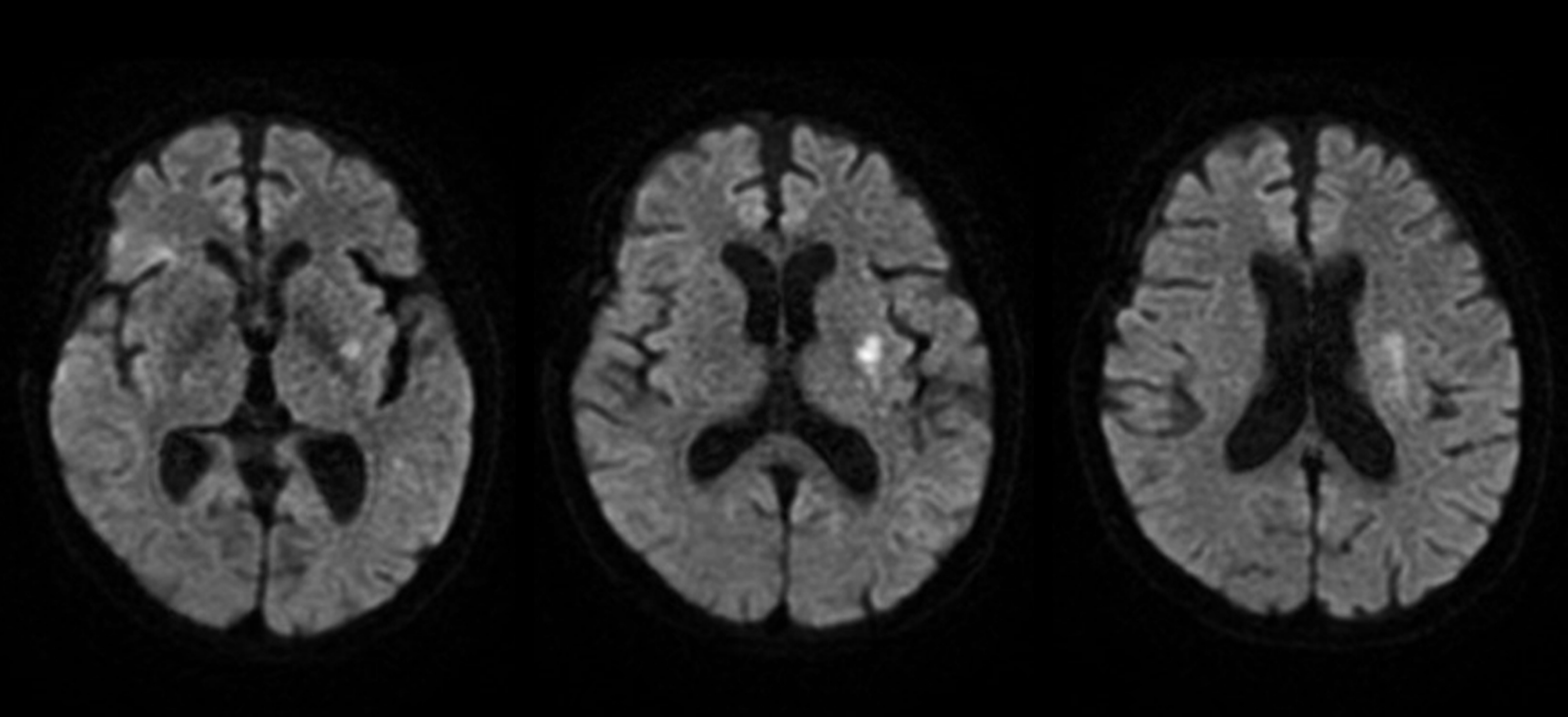
Brain MRI. Diffusion-weighted brain MRI showed ischemic stroke located between the posterior limb of the left internal capsule and the putamen. MRI: magnetic resonance imaging.

**Figure 2.**
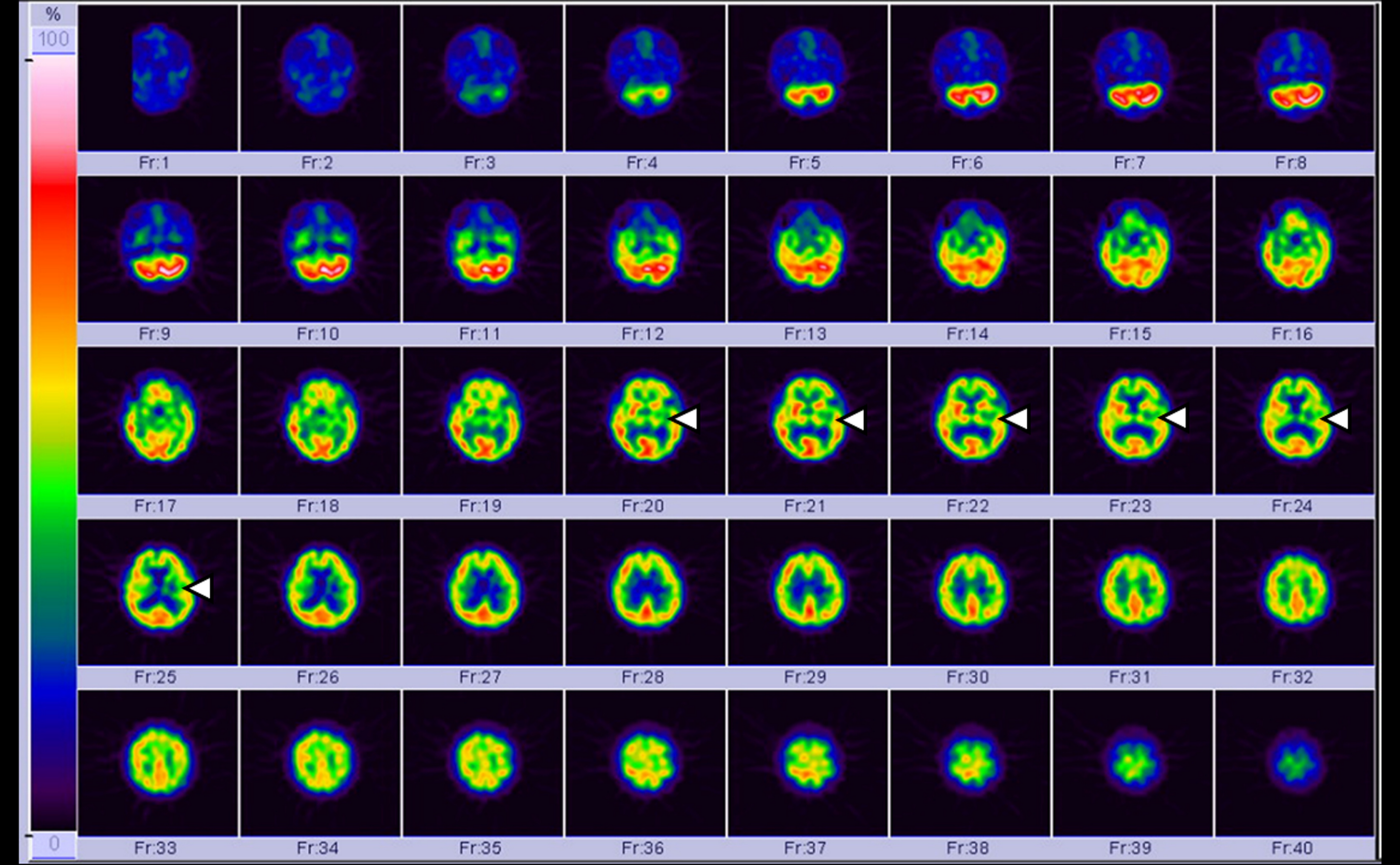
SPECT. SPECT using 99mTc-ethyl cysteinate dimer showed extensive hypoperfusion in the left lentiform nucleus, thalamus, and cerebral cortex. Arrow head: extensive hypoperfusion in the left lentiform nucleus and thalamus. SPECT: Single-photon emission computed tomography.

Unilateral asterixis is known to occur in subcortical brain lesions ^(1), (2)^. However, the exact pathophysiology of unilateral asterixis remains unclear ^(3)^. To address this, 99mTc-ECD SPECT, reflecting the blood-brain barrier breakdown or tissue damage, is an effective method to detect brain functional abnormalities ^(4)^.

In our previous report, iodine-123-labeled N-isopropyl-p-iodoamphetamine SPECT revealed extensive hypoperfusion in the basal ganglia, thalamus, and cerebral cortex in a patient who presented with unilateral asterixis ^(5)^. These cases further strengthen the notion that the pathophysiology of unilateral asterixis may involve dysfunction of the basal ganglia-thalamocortical circuits, as the circuits play critical roles in maintaining posture through continuous motor control.

## Article Information

### Author Contributions

Hideyuki Matsumoto, Toshiyuki Kakumoto, and Yasuhisa Sakurai were involved in the acquisition, analysis, or interpretation of data. Hideyuki Matsumoto and Naohiro Uchio drafted the manuscript. All authors contributed to the revision of the manuscript and approved the final version.

### Conflicts of Interest

None

### Institutional Review Board Approval

Not applicable.

### Informed Consent

The patient signed informed consent forms for academic use of the data.
